# Strategies for Advanced Oncolytic Virotherapy: Current Technology Innovations and Clinical Approaches

**DOI:** 10.3390/pharmaceutics14091811

**Published:** 2022-08-29

**Authors:** Qing Ji, Yuchen Wu, Andreas Albers, Meiyu Fang, Xu Qian

**Affiliations:** 1 Department of Rare and Head & Neck Oncology, Key Laboratory of Head & Neck Cancer Translational Research of Zhejiang Province, The Cancer Hospital of the University of Chinese Academy of Sciences (Zhejiang Cancer Hospital), Institute of Basic Medicine and Cancer (IBMC), Chinese Academy of Sciences, Hangzhou 310022, China; 2 Department of Clinical Laboratory, The Cancer Hospital of the University of Chinese Academy of Sciences (Zhejiang Cancer Hospital), Institute of Basic Medicine and Cancer (IBMC), Chinese Academy of Sciences, Hangzhou 310022, China; 3 Department of Otolaryngology, Head and Neck Surgery, Berlin Institute of Health, Charité-Universitätsmedizin Berlin, Corporate Member of Freie Universität Berlin, Humboldt-Universität zu Berlin, 13353 Berlin, Germany

**Keywords:** oncolytic viruses, nanotechnology, oncolytic virotherapy, immunotherapy, melanoma, head and neck cancer

## Abstract

Oncolytic virotherapy is a type of nanomedicine with a dual antitumor mechanism. Viruses are engineered to selectively infect and lyse cancer cells directly, leading to the release of soluble antigens which induce systemic antitumor immunity. Representative drug Talimogene laherparepvec has showed promising therapeutic effects in advanced melanoma, especially when combined with immune checkpoint inhibitors with moderate adverse effects. Diverse viruses like herpes simplex virus, adenovirus, vaccina virus, and so on could be engineered as vectors to express different transgenic payloads, vastly expanding the therapeutic potential of oncolytic virotherapy. A number of related clinical trials are under way which are mainly focusing on solid tumors. Studies about further optimizing the genome of oncolytic viruses or improving the delivering system are in the hotspot, indicating the future development of oncolytic virotherapy in the clinic. This review introduces the latest progress in clinical trials and pre-clinical studies as well as technology innovations directed at oncolytic viruses. The challenges and perspectives of oncolytic virotherapy towards clinical application are also discussed.

## 1. Introduction

Viruses have been gradually discovered for their antitumor effects since 1904, and a woman with acute leukemia showed clinical remission after a viral infection [1]. Therefore, many studies have been performed to discover the oncolytic ability of different types of viruses. It started with wild viruses, but the results were unsatisfactory due to the uncontrollability of wild viruses [2]. With the advent of genetic engineering by knockouts and/or knockins of certain genes in the viral genome can attenuate virulence and enhance tumor specificity of the virus, making the use of viruses in cancer therapeutics a reality [3]. In 2004, the first drug of the oncolytic virus Rigvir was approved to treat melanoma in Latvia, followed by Oncorine for head and neck cancer [4,5]. However, oncolytic virotherapy has not attracted much attention due to insufficient clinical evidence. The emergence of T-VEC, an oncolytic virus based on herpes simplex virus (HSV)-1, sparked renewed interest in oncolytic virotherapy [6]. Nowadays, a variety of oncolytic viruses have been developed, and those entered clinical trials showed promising results regarding safety and treatment efficacy [7].

Oncolytic viruses generally refer to viruses after genome engineering. Natural tropism and genetic targeting facilitate the specific selectivity of oncolytic viruses to the tumor cells. Briefly, oncolytic viruses inhibit tumors by directly infecting and lysing tumor cells and triggering a systemic immune attack. After infection, the virus starts its replication process, producing viral proteins, reducing cell function, stimulating oxidative stress states, and contributing to the activation of pathways being related to the autophagic processes. Lysis of cancer cells triggers the release of a variety of tumor-associated antigens (TAAs), which are taken up by the antigen-presenting cells (APC) to present tumor antigen information to naïve T cells in the lymph nodes. Thereafter, CD4+ and CD8+ T cells are stimulated, and at the same time, a series of cytokines are released that consequently lead to the migration and maturation of inflammatory cells. Antitumor immune response is activated. With the continuous oncolysis caused by the virus and immune attack, an increasing number of TAAs are released, thus generating more effector T cells and leading to a cascade of responses. Additionally, memory T cells recognizing TAAs distributed in the circulatory system will conduct immune tracking of distant tumors more than infected tumors [8,9]. However, current oncolytic virotherapy has not given full play to the ideal antitumor ability due to inadequate oncolytic ability, physical barriers, and antiviral host immunity during its delivery [10]. 

Considerable efforts have been made to improve the efficiency of oncolytic virotherapy. Strategies to combine oncolytic virus with conventional therapies, immune checkpoint inhibitors (ICIs), and even the chimeric antigen receptor (CAR)-engineered T-cell therapy, hold the hope for producing synergistic benefits [11,12]. Moreover, multiple functional elements have been introduced into the genome of oncolytic viruses to enhance their therapeutic effects. Additionally, a variety of delivery methods have been developed to concentrate the oncolytic viruses in tumor lesions more efficiently. In this review, we will introduce current applications of oncolytic viruses in the clinic, including approved drugs and representative clinical trials. Research on improving antitumor functions of oncolytic viruses and their delivery efficiency, will also be illustrated to discuss the progress and prospects of oncolytic virotherapy toward further clinical application.

## 2. Approved Oncolytic Viruses

Rigvir was the first approved oncolytic virus, based on ECHO-7 virus to treat melanoma in Latvia in 2004 [4]. Subsequently, a second oncolytic virus H101 (Oncorine) which is an oncolytic Ad5 with E1B-55kD deletion, was approved for nasopharyngeal carcinoma in 2005 in China [5]. In a multi-center, randomized and controlled phase III trial combining H101 with chemotherapy, the objective response rate (ORR) in squamous cell carcinoma of the head and neck (SCCHN) was 78.8%, compared with the 39.6% of control group (*p* < 0.001), demonstrating the immense anti-cancer efficacy of H101 combined with chemotherapy [13]. After its launch to the market, potential indications on liver cancer, pancreatic cancer, lung cancer, and malignant pleural effusion were also explored in clinical trials and have shown certain efficacy [5,14]. However, H101 had slightly impact on the market which might be due to the favorable efficacy of radiotherapy for nasopharyngeal carcinoma. 

Recently, talimogene laherparepvec (T-VEC) showed a significantly better ORR in unresectable stage IIIB-IV melanoma and was fast-tracked by the Food and Drug Administration (FDA) in 2015 [15]. T-VEC is a live, attenuated HSV-1 with deletions of the *ICP34.5* and *ICP47* and an insertion of a human granulocyte–macrophage colony-stimulating factor (*GM-CSF*) cassette [16]. Deletions of the *ICP34.5* and *ICP47* can improve tumor suppression effects and neuron safety of the virus, while the insertion of *GM-CSF* can promote local expression of GM-CSF and boost APC and trigger systemic antitumor immunity [16,17]. T-VEC is administered by intratumoral injection. Thus, melanoma is the most widely evaluated tumor due to its accessibility of lesions. In representative the OPTiM study, which was a randomized, phase III open-label clinical trial to evaluate T-VEC monotherapy compared with GM-CSF in patients with unresectable stage IIIB-IV melanoma, the ORR of T-VEC was significantly higher than that of GM-CSF (26.4% vs. 5.7%; *p* < 0.001) [15]. Thereafter, T-VEC was approved by the FDA, and a phase II clinical trial (ClinicalTrials.gov, NCT02211131) is currently being carried out to evaluate the efficacy of T-VEC as neoadjuvant therapy in melanoma [18]. According to the interim analysis, patients receiving neoadjuvant intratumoral injection of T-VEC have a two-year relapse-free survival (RFS) of 29.5% and a two-year overall survival (OS) of 88.9%, with an overall hazard ratio of 0.75 (80% CI = 0.58–0.96) and 0.49 (80% CI = 0.30–0.79), respectively, compared with those going straight to surgery [18]. Oncolytic virotherapy has attracted great attention again, and a series of clinical or preclinical studies have been actively conducted.

ICIs such as PD-1 inhibitors, have shown limited response rates in some cancer patients, especially those with poor immune infiltration in tumor lesions [19]. As mentioned above, oncolytic viruses can enhance antitumor effects by inducing systemic immunity. Studies have shown that T-VEC can promote immune infiltering systematically, turn the tumor microenvironment (TME) from ‘cold’ to ‘hot’, thus promote the efficacy of ICIs [20]. Preliminary results from phase I-II clinical trials demonstrated the benefits of combining T-VEC and ICIs in advanced melanoma. Whether combined with the PD-1 inhibitor pembrolizumab or the CTLA-4 inhibitor ipilimumab, the response rates of the combined therapy were significantly improved [20,21]. However, a phase III clinical trial of T-VEC in combination with pembrolizumab showed that the combination therapy did not significantly improve progression-free survival (PFS) or OS compared with the control group in unresectable stage IIIB–IVM1c melanoma [22]. Subgroup analysis of this trial suggested that melanoma patients with normal baseline levels of LDH and low tumor burden may benefit from this combination. It cannot be ruled out that the lack of improvement in PFS or OS may be partly related to the choice of evaluation criteria because the adoption of RECIST/iRECIST, which are criteria for systemic therapy in the assessment of local intervention, may not be accurate enough [23]. In other malignancies, such as SCCHN, sarcoma, and breast cancer, the efficacy of this combination therapy is also under investigation, and currently, it has shown tolerable safety profiles [24,25,26]. In addition to combining with ICIs, researchers have explored the combination of T-VEC with chemotherapy, radiotherapy, targeted therapy, or cell therapy [6,27,28]. Preoperative intratumoral T-VEC with concurrent external beam radiation therapy induced a 95% pathologic necrosis rate of 24% (7/29) in a phase IB/II clinical trial for soft tissue sarcomas [29]. In triple-negative breast cancer, 55% (5/9) of patients achieved RCB0 when treated with T-VEC and neoadjuvant chemotherapy [30]. The results of clinical trials on T-VEC combined with targeted therapy or cell therapy are still waiting to be reported. 

Teserpaturev/G47Δ (Delytact) is another approved oncolytic virus based on HSV-1 for glioma in Japan. G47Δ is oncolytic HSV-1 with the deletion of *ICP34.5* and *ICP47* genes, and a insertion of *lacZ* to inactivate the *ICP6* gene to further enhance the tumor specificity [27,28]. In a phase I/II study of G47Δ for recurrent or progressive glioblastoma, 13 patients were recruited and received repeated intratumoral injection of G47Δ (UMIN-CTR Clinical Trial Registry UMIN000002661). According to the latest results, G47Δ showed a better safety profile than T-VEC in the brain with a higher dose and was generally well tolerated. The median OS was 7.3 months and the one-year survival rate was 38.5%. Notably, three patients survived more than 46 months, including one who reached complete response (CR) and one who reached partial response (PR). The median PFS was eight days, possibly due to a sudden influx of immune infiltration towards tumor cells according to the histology of biopsy and the shrinkage of enlarged lesions after steroid administration, which suggest its potential to be combined with ICIs [31]. As seen in murine models, the combination of G47Δ and anti-CTLA-4 antibody has shown enhanced antitumor activity [32]. Recent published data from the phase II study show that for 19 patients recruited, the one-year survival rate after G47∆ initiation reached 84.2% in a higher total dose than that of the phase I/II study, and the median OS was 28.8 months from the initial surgery, which were better than the historical data (UMIN-CTR Clinical Trial Registry UMIN000015995) [32,33]. One patient reached PR, 18 patients reached stable disease (SD), and three patients were stable for more than three years after the last G47∆ administration, indicating that G47∆ could induce a long-term disease control [33]. The promising results indicate G47∆ is a favorable option for malignant glioma treatment.

## 3. Representative Oncolytic Viruses in Clinical Trials

Currently, a number of oncolytic viruses have entered clinical trials, and some preliminary data on safety and efficacy profiles have been reported. For example, HSV was used as the same vector as T-VEC and G47Δ, such as OrienX010 [34] and OH2 [29], and adenovirus was used as the same vector as Oncorine, for example CG0070 [35]. In fact, a variety of viruses can be used as genome engineering vectors for oncolytic viruses as long as there are no uncontrollable risks of serious toxicity. In this section, we summarize the status of oncolytic viruses under development with promising results in clinical trials (Table 1). Considering that some oncolytic viruses have not been further studied due to the early results of modest efficacy or other reasons, this section only introduces viruses with updated results from clinical trials in the last five years. 

Among recent clinical trials, the majority of oncolytic viruses under development are adenoviruses [35,36,37,38,39,40,41,42,43,44,45,46,47,48,49], followed by HSV, including HSV-1 and HSV-2 [34,50,51,52,53,54,55,56,57,58,59]. Others, such as reovirus, vaccina virus, and coxsackie virus, could also be used as vectors [60,61,62,63,64,65,66,67,68,69,70]. No data about phase III clinical trials of novel oncolytic virotherapy have been published in the past five years. Most of the reported data are from phase I clinical trials only with published abstracts in conferences, suggesting that the knowledge is limited to safety data thus far. Few treatment-related adverse effects above grade 4 are monitored, indicating that oncolytic virotherapy is generally safe and well tolerated. Preliminary efficacy data of some phase I clinical trials were published as it was seen most oncolytic virotherapies could bring certain benefits to patients with malignant tumors. Some phase II studies of oncolytic therapy have been published for their efficacy data. CG0070 is a replication-competent oncolytic adenovirus, intravesical of which yielded an overall 47% CR rate at six-month for patients with BCG-unresponsive non-muscle-invasive bladder cancer and 50% for patients with carcinoma-in-situ, comparable with other published trials with short follow-up [36]. OH2 is a genetically engineered oncolytic HSV-2 expressing GM-CSF. Among 40 patients with advanced solid tumors receiving single agent OH2 and 14 receiving OH2 in combination with HX008, four patients having rectal cancer or metastatic esophageal cancer achieved immune-PR, two in each group separately, demonstrating durable antitumor activity of OH2 in esophageal and rectal cancer [58]. ParvOryx is an oncolytic parvovirus administrated intravenously followed by intratumorally. There were 2 of 7 patients reaching PR (one unconfirmed) according to RECIST criteria. All patients showed T-cell responses to viral proteins, suggesting immune activation after administration of ParvOryx [69]. V937, also called CVA21, is an unmodified oncolytic coxsackievirus. In a phase II clinical trial of advanced melanoma recruiting 57 patients, the six-month PFS rate per immune-related RECIST was 38.6% (95% CI, 26.0 to 52.4) and durable response rate (DRR) was 21.1%. Regression was observed in both injected and noninjected lesions [71]. In summary, the current data are mainly from single arm phase I or phase II studies with small sample sizes, except Pelareorep which will be discussed in the next part. Subsequent clinical trials with expanded sample sizes are needed to further evaluate the efficacy of these novel oncolytic viruses. 

According to these clinical trials, oncolytic viruses are mainly administered as local injections combined with other therapies, such as chemotherapy or ICIs. As seen in earlier clinical trials, T-VEC is not as competitive as monotherapy [6]. Thus, it may be speculated that combination therapy would be an indispensable way for oncolytic viruses to play a critical role in the antitumor arena. Currently, the major method of oncolytic viruses’ administration is intratumoral injection or infusion in the cavity, leading to a local concentration of oncolytic viruses. Although oncolytic virotherapy does have a distant tumor suppressive effect, analyses of T-VEC and G47∆ have confirmed that effects in lesions receiving direct injection are significantly better than those in distal lesions [33,72]. Additionally, there is an unmet need to optimize delivery methods that can induce a stronger systemic tumor suppressive effect of oncolytic viruses. Notably, some oncolytic viruses, such as Celyvir, are administered systemically through autologous mesenchymal stem cells (MSCs), Pelareorep and Pexa-Vec through intravenous infusion, etc., [45,66,71]. Celyvir will be discussed in a later section. Pelareorep is a serotype 3 reovirus, intravenous delivery of which has showed good tolerance and encouraging efficacy in advanced pancreatic adenocarcinoma and melanoma [73,74]. However, no advantages of PFS were observed in randomized phase II studies for patients with colorectal cancer, breast cancer, and non-small cell lung cancer (NSCLC), while the OS of patients with metastatic breast cancer who received Pelareorep combined with paclitaxel was significantly prolonged when imbalanced baseline factors were excluded [75,76,77]. Pexa-Vec is a thymidine kinase gene-inactivated oncolytic vaccinia virus expressing human GM-CSF, which could be administrated both intratumorally and intravenously [78,79]. Most data of Pexa-Vec were published in 2015 or before and limited to safety data, except for liver cancer. The phase III study of intratumoral Pexa-Vec combined with sorafenib in liver cancer was terminated because of an unsatisfactory predicted endpoint in 2019 [80,81,82,83]. The most recent data of intravenous Pexa-Vec combined with durvalumab was published in the abstract of the 2020 American Society of Clinical Oncology, with an ORR of 7% in colorectal cancer [63]. In general, intravenous administration of oncolytic virus would be safe and well-tolerated with promising results, with the efficacy data mainly from single-arm phase I studies with small sample sizes. However, intratumoral administration showed significant improvement while intravenous administration did not in a meta-analysis [84]. It remains to be evaluated in future clinical trials for the efficacy of intravenous oncolytic viruses. Notably, lessons from clinical trials revealed limitations on the replication of some oncolytic viruses due to the existence of neutralizing antibodies in the blood, bio-barriers affecting the efficacy of delivery and rapid elimination of oncolytic viruses by activated immune system, etc. Thus, efforts to address these obstacles would be necessary to increase therapeutic concentrations of drugs with limited toxicity and improve the delivery efficacy.

**Table 1:** Overview of clinical trials with promising results of oncolytic viruses from 2017–2022.

Vector	Name	Indications	Interventions	Results	Phase	Registration Number
Adenovirus	CG0070	Bladder cancer	ip.	N = 45, Overall 6-month CR: 47%,	II	NCT02365818 [36]
			nivolumab + ip. (neoadjuvant followed by radio cystecmy)	N = 15, ORR = 54%	I	NCT04610671 [35]
			pembrolizumab + ip.	N = 35, CRR = 87.5% (16 evaluable)	II	NCT04387461 [37]
	ONCOS-102	Ovarian/Colorectal cancer	durvalumab + ip.	OC: N = 19, SD = 4; CRC: N = 36, SD = 9;	I/II	NCT02963831 [38]
	OBP-301	Hepatoma	it.	N = 20, SD = 7, PD = 11	I	NCT02293850 [39]
	DNX-2401	Glioma	it.	N = 25, reduction ≥ 95% = 3	I	NCT00805376 [40]
		Pediatric Glioma	Infusion in cerebella followed by radiotherapy	N = 12, PR = 3, SD = 8	I	NCT03178032 [85]
	Celyvir	Advanced Tumors	iv.	N = 16, SD = 2	I/II	NCT01844661 [41]
	VCN-01	Pancreatic adenocarcinoma	iv.	N = 26, ORR 50%	I	NCT02045602 [42]
	Enadenotucirev	Ovarian cancer	iv. + PTX/ip.	N = 38, ORR = 10%, * 6 discontinued treatments due to TRAE	I	NCT02028117 [44]
		Epithelial solid tumors	iv.	N = 61	I/II	NCT02028442 [43]
	Nsc-crad-s-pk7	Glioma	ip.	N = 12, mPFS = 9.1 m, mOS = 18.4 m	I	NCT03072134 [45]
	LOAd703	Pancreatic cancer	it. + nab-PTX + GEM	N = 22, ORR = 44%, DCR = 94% (18 evaluable)	I/II	NCT02705196 [46]
	Ad5-DS	Pancreatic cancer	it.	N = 11, PR = 2, SD = 2 (9 evaluable)	I	NCT02894944 [47]
	Ad5-yCD/*mut*TK(SR39)*rep*-hIL-12	Pancreatic cancer	it.	N = 12	I	NCT03281382 [48]
	CAN-2409	NSCLC	it. + ICIs ± chemotherapy	N = 28, PR = 4, SD = 8 (14 evaluable)	II	NCT04495153 [49]
Herpes simplex virus-1	VG161	Advanced solid tumors	it.	N = 3	I	ACTRN12620000244909 [50]
	HF-10	Melanoma.	it.+ ipilimumab	N = 46, ORR = 41%, DCR = 68%	II	NCT02272855 [51]
		Pancreatic cancer	it. + GEM + nab-PTX	N = 6, PR = 4, SD = 2	I	NCT03252808 [52]
	RP1	Skin cancer	it. + nivolumab	Melanoma: ORR = 13/36 (36.1%), Non-melanoma: ORR = 19/31 (61.3%)	II	NCT03767348 [53]
	OrienX010	Melanoma	it.	N = 26, ORR = 19.2%, DCR = 53.8%	Ib	CTR20140631/ CTR20150881 [34]
		Melanoma with liver metastases	it. + toripalimab	N = 23, ORR = 15%, DCR = 50% (20 evaluable)	I	NCT04206358 [86]
	Seprehvir	Refractory extracranial solid cancers	iv.	N = 9, SD = 2	I	NCT00931931 [54]
	G207	Pediatric Glioma	it.	N = 12, mOS = 12.2 m	I	NCT02457845 [55]
	T3011	Cutaneous/subcutaneous malignancies	it.	N = 8, SD = 5 (6 evaluable)	I/II	NCT04370587 [56]
	rQNestin	Glioma	it.	N = 30, mOS = 13.25 m	I	NCT03152318 [57]
Herpes simplex virus-2	OH2	Advanced solid tumors	it./it.+ HX008	OH2:PR = 2/54; OH2+ toripalimab: PR = 2/14	I/II	NCT03866525 [58]
		Melanoma	it.	N = 35, iPR/PR = 5, SD = 6	I/II	NCT04386967 [59]
Reovirus	Pelareorep	Pancreatic adenocarcinoma	iv. + pembrolizumab + chemotherapy	N = 11, SD = 3, PR = 1(10 evaluable)	I	NCT02620423 [60]
		Melanoma	iv. + CBP + PTX	N = 14, ORR = 21%, mPFS = 5.2 m, mOS = 10.9 m	II	NCT00984464 [74]
		Breast cancer	iv. + PTX vs. PTX	N = 36 vs. 38, mPFS = 3.78 m vs. 3.38 m, mOS = 17.4 m vs. 10.4 m	II	NCT01656538 [76]
Vaccinia virus	TG4023	Liver tumor	it. + flucytosine	N = 16	I	NCT00978107 [61]
	GL-ONC1	Ovarian cancer	ip.	N = 11	I/II	NCT02759588 [62]
	Pexa-Vec	Colorectal cancer	iv. + durvalumab/iv. + durvalumab + tremelimumab	N = 16, PR = 1(14 evaluable)	I/II	NCT03206073 [63]
Coxsackie virus	CVA21	Bladder Cancer	ip.	N = 15, CR = 1	I	NCT02316171 [64]
		Melanoma	it.	N = 57, 6-months PFS rate = 38.6%, DRR = 21.1%, ORR = 28.1%	II	NCT01227551/NCT01636882 [71]
			it. + ipilimumab	N = 13, ORR = 38.0%, DCR = 88%(8 evaluable)	I	NCT02307149 [65]
measles virus	MV-NIS	Urothelial carcinoma	iv.	N = 8, pCR = 2	I	NCT03171493 [66]
Poliovirus	PVSRIPO	Melanoma	it.	N = 12, ORR = 33%, pCR = 2	I	NCT03712358 [67]
		Glioblastoma	it.	N = 61, a plateau of 21% at 24 months	I	NCT01491893 [68]
Parvovirus	ParvOryx	Pancreatic Adenocarcinoma	iv.	N = 7, PR = 1	I/II	NCT02653313 [69]
Vesicular stomatitis virus	VSV-IFNβ-NIS	Refractory solid tumors	iv.	N = 18	I	NCT02923466 [70]

* The safety results of other clinical trials are all well tolerated. ip.: intracavity perfusion; it.: intratumoral injection; iv.: intravenous injection; ICIs: immune checkpoint inhibitors; PTX: paclitaxel; CBP: carboplatin; GEM: gemcitabine; CR: complete response; CRR: complete response rate; PR: partial response; pCR: pathological complete response; pPR: pathological partial response; iPR: immune partial response; ORR: objective response rate; SD: stable disease; PD: progressive disease; DCR: disease control rate; DRR: partial or complete response for ≥ 6 months; OS: overall survival; TRAE: treatment related adverse event; CRC: colorectal cancer.

## 4. Genome-Engineering Approaches to Improve Therapeutic Effects of Oncolytic Viruses

**Figure 1:**
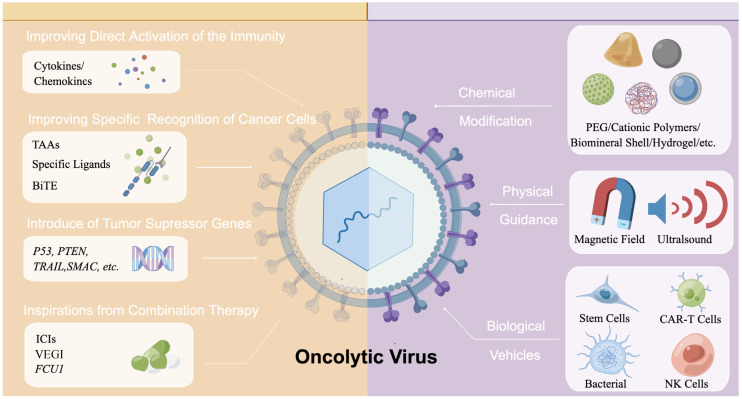
Strategies for improving oncolytic virotherapy. Strategies are divided into two main categories: improvements of genome engineering and of the delivery system. In terms of the genome engineering (**left**), various functional elements can be designed into the genome of oncolytic virus, including those can activate the immune system directly, specific recognition of cancer cells, tumor-suppressor genes, and inspirations from other therapies. In terms of the delivery system (**right**), multiple nanomaterials could be used as chemical modifications to the capsule of viruses. In addition, physical technology such as magnetic fields and ultrasound could also help in the guidance of oncolytic viruses. Stem cells, CAR-T cells, and even bacteria could be used as vehicles to transport oncolytic viruses. TAAs: Tumor-associated antigens; BiTE: Bispecific T cell engager; ICIs: Immune checkpoint inhibitors; VEGI: vascular endothelial cell growth inhibitor; PEG: poly(ethylene) glycol; CAR: chimeric antigen receptor; NK cells: natural killer cells. The figure is drawn by FigDraw.

The immune system could be a double-edged sword for oncolytic therapy. On the one hand, the antiviral immune response must be minimized to avoid inhibiting viral replication and promoting viral transmission within the tumor. On the other hand, it requires mobilization of innate and adaptive immune responses and monitoring of the immunosuppressive TME to maximize the antitumor effects. To balance such responses of the immune system, many efforts have been made to optimize current oncolytic viruses or develop new viruses to enhance the stimulation of host immune responses to tumor cells without triggering quick clearance of oncolytic viruses (Figure 1 & Table 2). For example, modifications have been made to previously established oncolytic viruses, such as the deletions of *ICP34.5* and *ICP47* in T-VEC [6]. Moreover, cytokines or chemokines can be introduced into the genome to further enhance the therapeutic effects of oncolytic viruses. For example, *GM-CSF*, an immune-related cytokine, can increase the activation of APC and trigger a systemic antitumor immune response [15,34]. Additionally, TAAs, immune-related ligands or bispecific T-cell engager (BiTE) antibodies can also modify the oncolytic viruses. Inspired by combination therapy, insertion into the genome of oncolytic viruses with functional elements related to other antitumor therapies has also been studied.

### 4.1. Introducing Immune Activating Cytokines/Chemokines Directly

Activation of systemic immunity is one of the key antitumor mechanisms of oncolytic virus. For this reason, *GM-CSF* is inserted into T-VEC, OH2, etc., to further enhance the ability of oncolytic virus to induce immunity against tumor, which has shown their great benefits in clinical trials [15,58]. In addition to GM-CSF, cytokines such as TNF, IFN-α/β, IL-12, IL-7, or chemokines such as CXCL9 and CXCL10 have been tested as immune enhancers in the genomes of oncolytic viruses, and some of them have shown their abilities to promote antitumor activities in preclinical tumor models and clinical trials [72]. For example, interleukin-12 (IL-12) is a potent anticancer agent that promotes T-helper 1 (Th1) differentiation, facilitates T-cell-mediated killing of cancer cells, and inhibits tumor angiogenesis [100]. A variety of IL-12-expressing oncolytic viruses have been genetically engineered, including herpes simplex virus, adenoviruses, and measles virus [101,102,103]. The safety and efficacy of IL-12 have been verified in preclinical tumor models [104,105,106]. Currently, IL-12-expressing HSV (M032, ClinicalTrials.gov Identifier: NCT02062827) and vaccina virus (ASP9801, ClinicalTrials.gov Identifier: NCT03954067) are being evaluated in phase I clinical trials. Another cytokine that is widely considered is TNF-α, which is also a major proinflammatory cytokine [107]. Oncolytic viruses with TNF-α can eradicate tumors and induce antitumor T-cell responses in various tumor models [87]. In breast tumors, it was found that TNF-α-armed oncolytic vesicular stomatitis virus (VSV) could improve survival [108]. Other cytokines, including IFN-α/β, IL-7, IL-15, IL-18, IL-23 and IL-24, have also shown certain effects [72]. 

Chemokines are small secreted chemotactic cytokines that can mediate the migration of immune cells. When stimulated by chemokines, different immune cell subsets migrate into the TME and regulate antitumor immune responses in a spatiotemporal manner [109]. Intratumoral delivery of CXCL11-expressing vaccina virus could induce an aggregation of adoptive T-cells in tumor tissues and prolong the survivals of tumor-bearing mice [88,89]. However, an oncolytic VSV engineered to express CXCL9 did not show enhanced antitumor activity compare to the control virus [110], which indicates that careful selection or combination of these cytokines is needed to develop more efficient oncolytic viruses. NG-641, an oncolytic adenovirus expressing CXCL9, CXCL10, and IFN, is currently undergoing a phase I clinical trial to evaluate its safety and tolerability (ClinicalTrials.gov Identifier: NCT04053283). The results are yet to be reported.

### 4.2. Introducing Elements to Improve Specific Recognition and Immunity against Tumors

One other design of oncolytic virotherapy is introducing tumor-specific antigens or acting molecules that would facilitate the identification of oncolytic virus and induce systemic immunity. TAAs are self-antigens expressed by tumor cells that are commonly used in the construction of tumor vaccines to induce a specific and sustained systemic antitumor response and have been verified in multiple animal models [111]. The introduction of TAA into oncolytic viruses is a promising genetic modification approach that can further enhance the induced antitumor immunity. Recombinant vaccine strain-derived measles virus carrying claudin-6 significantly inhibited metastasis and increased the therapeutic efficacy in in vivo tumor models being established with syngeneic B16-hCD46/mCLDN6 murine melanoma cells [90]. A novel platform using adenovirus 5 vectors with three TAAs targeted, including prostate-specific antigen (PSA), brachyury, and MUC-1, has been developed for a cancer vaccine and is well tolerated in the phase I study of metastatic castration-resistant prostate cancer [91]. 

In the era of immunotherapy, costimulatory molecules such as CD28, B7.1, and intercellular adhesion molecule 1, or ICIs such as PD-1, CTLA-4, TIM3, and LAG3 have attracted significant attention in cancer therapeutics [65,112,113]. Oncolytic virotherapy combined with ICIs has shown promising prospects in clinical trials [20,21], prompting further developments of engineered oncolytic viruses with ICIs, which will be discussed later. Engineering other ligands with the genome of oncolytic viruses has also been investigated. A recombinant Newcastle disease virus expressing the inducible costimulator was found to enhance T-cell infiltration in both virus-injected and distant tumors in mice, and induce rejection of both tumors [114]. HSV-1-based oncolytic viruses encoding OX40 L and IL-12 can transform the infected tumor cells to artificial APC, induce complete tumor regression in patient-derived xenograft and syngeneic mouse tumor models, and elicit antitumor immune memory [92]. Currently, an oncolytic adenovirus (LOAd703) expressing CD40 L and 4-1BBL has shown potential for immune activations in various tumor models and has been investigated in clinical trials [93].

BiTE, a recombinant bispecific protein, has two linked single-chain fragment variables (scFvs) derived from two individual antibodies by which can target both a cell-surface molecule on T cells and antigen on the surface of malignant cells [115,116]. However, the on-target off-tumor activity might lead to dose-limiting toxicities of BiTE due to the design for dual effect of oncolytic viruses that can cobind tumor cells and T cells simultaneously, restrict their delivery to the tumor site. Using malignant serous effusion for infection of the BiTE-expressing EnAdenotucirev leads to activation of endogenous T cells to inhibit tumor cells [117]. In tumor-bearing mice, oncolytic measles viruses encoding BiTE could increase T-cell infiltration in the TME and induce antitumor immunity without intolerable toxicity [94]. Recently, a novel strategy of oncolytic herpesvirus (oHSV-1) expressing PD-L1 BiTE that can enhance T-cell activation and the release of cytokines was found to has the potential to treat immune ‘cold’ tumors [118].

### 4.3. Introducing Tumor Suppressor Genes Associated with Tumor Cell Apoptosis

Dysregulation of tumor suppressor genes can lead to the formation of cancer [119]. Engineering tumor suppressor genes into oncolytic viruses could facilitate tumor apoptosis and enhance treatment efficacy, i.e., *P53* and *PTEN* [96,120]. Various viruses, including adenovirus, herpesvirus, vesicular stomatitis virus, and Newcastle disease virus, have been used as vectors to carry the *P53* gene in cancer therapeutics [95,121,122,123,124]. Some of them have been applied in clinical trials with the safety data being published. However, most results were from phase I clinical trials and published years ago, lacking recent updates on further clinical trials [120]. An oncolytic HSV co-expressing *PTEN* was also shown to have the potential to boost immune responses against tumor and led to tumor rejection and long-term survival in mice bearing intracranial tumors [96]. Additionally, oncolytic viruses encoding other tumor-suppressor genes such as *TRAIL* and *SMAC* can also lead to tumor-cell apoptosis [125,126,127].

### 4.4. Introducing Functional Elements Being Related to Other Antitumor Therapies

Combining oncolytic virotherapy with other antitumor therapies is promising. Other than direct combinations, introducing functional elements related to ICIs, antiangiogenic drugs, or cytotoxic chemicals into oncolytic viruses might strengthen therapeutic efficacy due to a more convenient administration, widened therapeutic window and less toxity. For example, oncolytic viruses in combination with ICIs have impressive effects in clinical trials [26,128]. As mentioned above, oncolytic viruses have been engineered to directly express ICIs such as PD-1/PD-L1 inhibitors, which have shown certain effects in murine melanoma models. Newcastle disease viruses engineered to express anti-PD-1/PD-L1 elements induced tumor control and benefited survival in highly aggressive B16-F10 murine melanoma models [97]. Expression of a PD-1 inhibitor in HSV-1 could also induce obvious antitumor effects in murine melanoma models [129]. In addition, antiangiogenic targeted therapy or traditional chemotherapy, has also been integrated into oncolytic virotherapy. For example, antiangiogenic drugs such as bevacizumab, as one of the classical antitumor drugs, play an important role in cancer therapeutics [130]. Oncolytic viruses encoding antiangiogenic transgenes have been explored for their antitumor ability. An adenovirus with E1B 55 kDa gene deletion and vascular endothelial cell growth inhibitor insertion was constructed and showed anticancer therapeutic potential in athymic nude mice bearing human cervical and colorectal tumor xenografts by inhibiting endothelial cell proliferation, tube formation, and angiogenesis [98].

The integration of chemotherapy with genome engineering of oncolytic viruses is one of the cornerstones in antitumor therapy. Cytosine deaminase can convert nontoxic 5-fluorocytosine to the highly toxic 5-fluorouracil, which is an indispensable chemotherapy agent in the treatment of gastrointestinal cancer and others. A thymidine kinase gene-deleted vaccinia virus that expressed the fusion suicide gene *FCU1* derived from the yeast cytosine deaminase and uracil phosphoribosyltransferase genes could produce substantial tumor growth retardation in the presence of systemically administered prodrug 5-fluorocytosine, representing a potentially efficient means for oncolytic therapy [99]. Similarly, oncolytic viruses carrying other genes such as *HSV-TK* that can convert ganciclovir to the toxic ganciclovir phosphate, or genes encoding nitroreductase and cytochrome P450, have shown antitumor therapeutic potential in preclinical models [131,132,133].

## 5. Innovations for Oncolytic Viruses Delivery

To date, the common delivery method of oncolytic viruses is intratumoral injection, in which oncolytic viruses are injected directly into the tumor lesion, which is suitable for superficial lesions such as cutaneous melanoma. In addition, with the guidance of ultrasound, lesions in viscera such as liver and kidney could also be injected directly, but this process is comparatively complicated, and risks such as infection and bleeding cannot be avoided. Another common method is intravenous delivery, which ideally can deliver oncolytic viruses to any location needed and possesses great advantages of convenience and safety. However, due to the degradation in circulation, rapid clearance by the antiviral immunity effect, sequestration in nontarget organs or physical barriers such as the endothelial layer and the dense extracellular matrix, intravenous administration of oncolytic virotherapy has limited success [134,135]. In addition, infusion of oncolytic viruses in thoracic, abdominal, bladder, and other cavities in the body has been reported in some phase I clinicals, but the indications are relatively limited [37,38,40]. In conclusion, although oncolytic viruses can induce a systemic immune response and lead to tumor regression as seen in clinical trials, the lower response of distant lesions compared to injected lesions would be critical in oncolytic therapy [136]. Researchers are trying to utilize chemical modifications or biological vectors to further improve the efficiency of oncolytic virus delivery (Figure 1).

### 5.1. Chemical and Physical Methods Assisting the Delivery

Nanomaterials such as poly(ethylene) glycol (PEG) and cationic polymers are designed to enhance the systemic delivery of oncolytic viruses. PEG is a hydrophilic molecule that has been widely researched as a delivery vehicle in biomedical applications with negligible cytotoxicity [137,138]. PEG protected oncolytic viruses from the immune system at a sufficient therapeutic level of infection. Coating oncolytic adenoviruses with PEG reduces the transduction of hepatocytes and hepatotoxicity after intravenous administration. The antitumor efficacy of PEGylated oncolytic adenoviruses was increased in hepatocellular carcinoma xenografts where a longer tumor-free survival was observed [139]. Cholesterol-PEG can significantly reduce the binding of an anti-vaccinia virus neutralizing antibody [140]. Cationic polymers contain a main chain with positively charged amine groups. Oncolytic adenoviruses and polymer complexes with net cationic surface charge promoted cellular uptake and transgene expression, while it is worth remembering that the highly positive surface charge could also lead to increased cytotoxicity [141,142]. Notably, chemical modification might bring an additional benefit. Coating the oncolytic adenovirus with a biomineral shell composed of manganese carbonates (MnCaCs) could realize the possibility of real-time monitoring with the help of Mn^2+^ and the increased O_2_ from endogenous H_2_O_2_ by T1 modal magnetic resonance imaging (MRI) and photoacoustic imaging (PAI), and the biomineral shell could also provide protection from removal of the host immune system at the same time [143]. This approach is promising for testing in clinical trials.

Another promising nanomaterial to deliver the oncolytic virus is the hydrogel that is formed by self-assembly or crosslinking of polymers with a highly porous and hydratable structure, thus enabling a high concentration of the viruses in the target lesions and protecting them from rapid clearance by the immune system [144]. Recently, a gelatin-based hydrogel codelivering an oncolytic adenovirus armed with IL-12 and IL-15 and CIK cells was developed, which can induce a durable antitumor immune response with a single administration, and displays a more potent therapeutic effect in established lung and intestinal cancers [145]. Additionally, vehicles such as poly[N-(2-hydroxypropyl) methacrylamide] (PHPMA) have also been studied for delivering oncolytic viruses [146,147]. Most of these studies are limited to preclinical studies, and further exploration in clinical trials is warranted.

Some physical methods are also being utilized for the delivery of oncolytic viruses. For example, it has been demonstrated that magnetic nanoparticle-encapsulated oncolytic viruses resulted in an increased infection and a better tumor suppression effect with the aid of a magnetic field [148,149,150]. Recently, a magnetic targeting strategy has also been combined with cells to form cell robots carrying on with oncolytic virus capable of tumor-selective binding and killing [151]. Fe_3_O_4_ nanoparticles are asymmetrically modified on the surface of oncolytic adenovirus-loaded cell robots to fabricate oncolytic viruses with a magnetic response. Under magnetic control, the cell robots are able to perform directional migration and have prolonged retention in the mouse bladder [151]. In addition, ultrasound could also assist in the delivery of the oncolytic virus. Acoustic cavitation has been found to play a potentially key role in both achieving targeted drug release and enhanced extravasation [152]. Oncolytic viruses can be propelled hundreds of microns with the help of inertial cavitation induced by the ultrasound. A polymeric cup formulation that provides the nuclei for instigation of sustained inertial cavitation events within tumors increased the activity of vaccinia viruses significantly and induced a significant retardation of tumor growth [153]. 

### 5.2. Biological Vehicles to Deliver Oncolytic Viruses

With the remarkable clinical success of CAR-T therapy in leukemias and lymphomas [154], adoptive T-cell therapies (ACTs) have attracted research interest. ACTs involve the infusion of tumor-specific cytotoxic T cells, thus exerting more antitumor immunity specifically [155]. Oncolytic viruses combined with ACTs can also be delivered into the tumor lesions, which has been explored both preclinically and clinically. It was found in patients given intravenous injections of reovirus that all viral particles detected in the blood were associated with peripheral blood mononuclear cells in patients with colorectal cancer, suggesting that cells can be used as carriers of oncolytic virus [156].

A variety of cell types have been demonstrated as potential carriers of oncolytic viruses, among which stem cells are an important candidate due to their nature of an inherent tropism toward invasive malignancies [157,158]. Stem cells from various sources including MSCs and neural stem cells (NSCs) have been tested extensively for their effectiveness. MSCs infected with engineered adenoviruses expressing IL-12 and PD-L1 inhibitors showed the ability to deliver and produce functional viruses to infect and lyse lung tumor cells and stimulate antitumor activity by releasing IL-12 and PD-L1 inhibitors [159]. Systemic administration of hepatocellular carcinoma-targeted oncolytic adenovirus-infected MSCs resulted in a high level of virion accumulation in the tumor lesion and inhibit tumor growth at a low initial viral infecting dose [160]. No grade 2–5 toxicities were reported in a phase I clinical trial for Celyvir, which is a kind of autologous MSCs carrying an oncolytic adenovirus. In this study, two patients with neuroblastoma showed disease stabilization, including one who continued on treatment for up to six additional weeks [41]. Although 18 of 34 recruited patients were excluded mainly because of rapid disease progression before Celyvir treatment, the results from this phase I clinical trial indicate the potential use of this stem cell carrier for further clinical investigation. The results of a phase I clinical trial using NSCs as oncolytic virus delivery vectors were also published [45]. Twelve patients with newly diagnosed malignant gliomas were recruited, of whom the median follow-up was 18 months, the median PFS was 9.1 months and the median OS was 18.4 months. Treatment was well-tolerated as no formal dose-limiting toxicity was reached [45]. Delivering an oncolytic virus by NSCs was feasible and safe. Natural killer (NK) cells are another option for tumor-targeting delivery of oncolytic virus due to their well-established homing ability to accumulate in tumor lesions and their highly cytotoxic immune effects [161,162]. Ad@NK is generated in which NK cells act as bioreactors and shelters for the highly efficient systemic tumor-targeted delivery of adenoviruses. As feedback, adenovirus infection offers NK cells an enhanced antitumor immunity by activating type I interferon signaling. Ad@NKs were proven to have excellent antitumor and antimetastatic functions both in vitro and in vivo [163]. CAR-T therapy and oncolytic viruses are also combined to increase the efficacy in solid tumors. In vitro preloading of CAR-T cells with oncolytic vesicular stomatitis virus or reovirus allows a further in vivo expansion and reactivation of T cells and leads to a prolonged survival of mice with subcutaneous melanoma and intracranial glioma [164].

Recently, bacteria have also been found to be able to assist in oncolytic virotherapy. It have shown that bacteria have a much higher homing capacity than that of oncolytic viruses, while there are also limited effectiveness and dose-dependent toxicity [165,166,167]. Sun, etc., proposed the concept of bacteria-assisted targeting of oncolytic viruses to tumors [168]. Liposome-cloaked oncolytic adenoviruses are conjugated onto tumor-homing Escherichia coli BL21. Compared with intravenously injected bare oncolytic adenoviruses, the enrichment of bacteria-assisted viruses in NSCLC was potentiated by more than 170-fold. Enhanced antitumor immunity was also demonstrated in vivo [168]. This suggests that bacteria with a higher tumor-homing capacity can be a potent platform for delivering oncolytic viruses.

## 6. Challenges and Perspectives

Oncolytic viruses are a novel immunotherapy, and numerous studies have been carried out preclinically and clinically, demonstrating the great potential of oncolytic virotherapy. However, some unsatisfactory data regarding clinical efficacy suggest that there are certain limitations that still need to be addressed. Patients with extensive small-cell lung cancer receiving platinum-based chemotherapy do not benefit from the oncolytic virus NTX-010. There was no difference in PFS between the NTX-010 and the control group [169]. The phase III trial of Pexa-Vec investigating the benefit of oncolytic virus combined with sorafenib was stopped recently because the primary endpoint of OS did not appear to be significantly different (ClinicalTrials.gov Identifier: NCT02562755). Although the combination of oncolytic reovirus Pelareorep with FOLFOX6/bevacizumab was well tolerated and led to an increased ORR, the PFS was inferior [75]. Even for T-VEC, which has been approved and widely recognized, the efficacy of its monotherapy is not superior to that of traditional chemotherapy or ICIs in melanoma. Further analysis found that the treatment of oncolytic virotherapy is better in the injected lesions but poor in distant lesions [136]. Recently, G47Δ brings great advances in the treatment of recurrent or progressive glioblastoma. It was also observed that the treated lesions are better controlled than distant ones. However, repeated burr hole surgeries to inject G47Δ into glioma lesions would be relatively painful for patients [31,33].

These limitations regarding treatment efficacy in clinical trials may be due to biological barriers, tumor heterogeneity, and the immunosuppressive TME [10]. Biological barriers constituted by the endodermis and interstitial hypertension in tumor due to abnormal lymphatic networks, vascular hyperpermeability, and dense extracellular matrix would also limit the viral invasion [170,171]. The preexisting or treatment-induced neutralizing antiviral antibodies in the host block the replication of oncolytic viruses and thus attenuate their ability to lyse tumor cells [172]. In addition, sequestration may occur via uptake to nontarget organs such as the spleen and liver [135]. Because the TME is characterized by immunosuppression for the convenience of tumor cells to escape the immune surveillance, especially a ‘cold’ TME, it is difficult for oncolytic viruses to recruit immune cells to kill tumors in such an immunosuppressive microenvironment [10]. 

Several approaches have been proposed to enhance oncolytic virotherapy. Combining with other therapies, such as chemotherapy or ICIs, is common sense and has been discussed in the above section of T-VEC. In addition, the combination with CAR-T cells is also of research interest [173]. Currently, genome engineering of oncolytic viruses with PD-L1-related elements has shown extremely significant therapeutic advantages [118]. Oncolytic viruses can also integrate other functional elements through genome engineering to exert a synergistic antitumor effect. However, numerous attempts have been made in the genome engineering of oncolytic viruses, with few results from phase III clinical trials have been published, most of which are limited to early clinical trials. It still needs to be validated carefully that which regimen of combination therapy would yield improved treatment efficacy with satisfactory side effects. 

Efforts to improve the specificity and oncolytic abilities of the virus itself should also be considered. One possibility is the selection of suitable viral vectors. It is a foundation to select an appropriate vector to develop oncolytic virotherapy which determines the efficacy of elements’ expression, interactions with the TME and additional payloads during the delivery. Adenovirus and HSV are the most common, and many others, such as the vaccinia virus, reovirus, and measles virus, have all entered in clinical trials and been approved for safety and preliminary efficacy. Selection of viral vectors with a comprehensive understanding of their characteristics and, on the basis of that, reasonable modifications and methods for administration, could produce better therapeutic effects. As an example, vaccina virus is usually applied in intravenous routes due to its wide tropism and the particular form of extracellular enveloped virion which can escape from neutralizing antibodies and complement clearance, resulting in the delivery of multiple payloads without compromises of replication and cytotoxicity [174,175].

The other is improvement of methods to deliver the oncolytic virus to tumor lesions more effectively, to maximally exert its antitumor effects. Either endogenous cells or exogenous bacteria have been used as transporters for oncolytic viruses and shown promising results. However, there are obvious limitations of cell carriers because of the need for ex vivo infection of cells and the manufacturing cost to bring two very complex biologic therapeutics into one product [3]. As mentioned before, novel chemical modifications and materials such as PEG, cationic polymers, hydrogels, and the use of ultrasound could also be applied to enhance the systemic delivery of oncolytic viruses in preclinical studies. As a result, although oncolytic viruses in the clinic are currently mainly delivered directly through intratumor/venous, injection, or intracavity perfusion, an increasing number of studies on the method innovations of delivery predict more efficient and less toxic ways to deliver oncolytic viruses in the future, which would greatly broaden the prospects of oncolytic virotherapy.

## 7. Conclusions

Oncolytic virotherapy is a promising immunotherapy for malignancies and has also shown promising therapeutic potential in clinical trials. As we move forward with transforming research to clinical application, sophisticated subgroup analysis of clinical trials to identify the appropriate therapeutic intervention time of oncolytic viruses, as well as to understand the underlying mechanisms for its interactions with tumor and the host, would help us to develop more powerful products toward more effective treatments. Additionally, response criteria currently in use for oncolytic virotherapy are those being established in the solid tumor guidelines (RECIST) regardless of new patterns of response that have been observed. Thus, a new concept of criteria should be assessed for future clinical trials. Moreover, the efforts to define biomarkers that can stratify patient populations would also be important to examine the response to new oncolytic virotherapy strategies. Importantly, the advanced nanotechnology innovation would also bring oncolytic virotherapy to a new landscape.

## Figures and Tables

**Table 2:** Examples of genome engineering categories for oncolytic virus.

Genetic Modification	Transgenes	Virus	Models	Main Results
Cytokines	GM-CSF	HSV-1	Clinical trials of Melanoma etc.	Approved by the FDA [15]
	IL-12 (NCT02062827)	HSV-1	Phase I study of glioma	Not published
	TNF-α	Vesicular stomatitis virus	Refractory orthotopic mouse model of mammary cancer (combined with SMC)	Prolonged survival and slower tumor growth [87]
Chemokines	CXCL11	Vaccina virus	intraperitoneal mouse models of mesothelioma and colon cancer	Induction of systemic antitumor immunity and better survival [88,89]
	CXCL9, CXCL10, IFN (NCT04053283)	Adenovirus	Phase I clinical trial of epithelial tumors	Not published
Tumor specific antigens	Claudin-6	Measles virus	B16-hCD46/mCLDN6 melanoma mouse models	Inhibit metastasis and increase the therapeutic efficacy [90]
	PSA, brachyury, MUC-1	Vaccina virus	Phase I clinical trial of castration-resistant prostate cancer	Well tolerated [91]
Cell surface molecules	OX40L (and IL-12)	HSV-1	Patient-derived oral xenograft and syngeneic colon and pancreatic mouse tumor models	Complete tumor regression [92]
	CD40L, 4-1BBL	Adenovirus	In vivo xenograft mouse models of pancreatic cancer	Reduced tumor burden [93]
BiTE	α-CD3-VH/L,α-TAA-VH/L	Measles virus	Syngeneic and xenograft mouse models of melanoma and colorectal cancer	Protective antitumor immunity and prolonged survival [94]
Tumor-suppressor genes	P53	Adenovirus	EH-GB1 xenografts mouse model	Inhibit tumor growth [95]
	PTEN	HSV-1	Intracranial tumor mouse model	Long-term survival and prime anti-cancer T-cell immunity [96]
Immune checkpoint inhibitors	Anti PD-1/PD-L1 (and IL-12)	Newcastle disease viruses	B16-F10 melanoma mouse model	Induce tumor control and survival benefits [97]
Anti-angiogenic transgenes	VEGI-251(and E1B 55 kDa deletion)	Adenovirus	Human cervical and colorectal tumor xenografts mouse model	Inhibit tumor growth [98]
Genes associated with cytotoxic medicine	FCU1	Vaccina virus	Orthotopic liver metastasis of colon cancer xenografts mouse model (combined with SMC)	Substantial tumor growth retardation [99]

HSV: herpes simplex virus; SMC: smac mimetic compounds; PSA: prostate-specific antigen; BiTE: bispecific T-cell engager; α-CD3-VH/L: anti-murine/human-CD3 variable heavy/light domain; α-TAA-VH/L: antitumor-associated antigen (CD20/CEA) variable heavy/light domain; VEGI: vascular endothelial cell growth inhibitor.

## Data Availability

Not applicable.
